# Indirect Annuloplasty to Treat Functional Mitral Regurgitation: Current Results and Future Perspectives

**DOI:** 10.3389/fcvm.2019.00060

**Published:** 2019-05-17

**Authors:** Tiffany Patterson, Heath Adams, Christopher Allen, Ronak Rajani, Bernard Prendergast, Simon Redwood

**Affiliations:** ^1^Cardiovascular, King's College London, St. Thomas Hospital, London, United Kingdom; ^2^Cardiovascular, Guy's and St. Thomas' NHS Foundation Trust, London, United Kingdom

**Keywords:** mitral regurgitation, annuloplasty, transcatheter, functional mitral regurgitation, indirect annuloplasty

## Abstract

The incidence of mitral regurgitation (MR) is approximately 1.7% in the developed world, and this increases to more than 10% in patients aged over 75 years. Functional (or secondary) mitral regurgitation (FMR) is defined as poor leaflet coaptation and tethering secondary to either ischemic or non-ischemic left ventricular (LV) dysfunction and dilatation. FMR is more common than degenerative (or primary) MR and is associated with significantly worse outcomes in patients with heart failure, post myocardial infarction and following coronary artery bypass graft surgery. Patients with severe degenerative MR have excellent outcomes with surgical repair, however the benefits of surgery in FMR are less clear. Although annuloplasty is associated with a lower operative mortality compared to replacement, the recurrence rate of mitral regurgitation is high in patients with FMR and neither surgical repair or replacement have been shown to reduce hospitalisation or death in FMR. Furthermore, nearly half of patients are deemed too high risk for surgery and therefore most patients are managed conservatively and there remains an unmet clinical need. Transcatheter mitral valve interventions are an emerging alternative for those at high surgical risk. This mini review focuses on indirect mitral annuloplasty: anatomical considerations, patient selection, current devices, implantation techniques and the associated clinical outcome data.

## Background

The incidence of mitral regurgitation (MR) is ~1.7% in the developed world, and this increases to more than 10% in patients aged over 75 years (1). Functional (or secondary) mitral regurgitation (FMR) is defined as poor leaflet coaptation and tethering secondary to either ischemic or non-ischemic left ventricular (LV) dysfunction and dilatation. FMR is more common than degenerative (or primary) MR and is associated with significantly worse outcomes in patients with heart failure, post myocardial infarction and following coronary artery bypass graft surgery (2–4). Patients with severe degenerative MR have excellent outcomes with surgical repair, however the benefits of surgery in FMR are less clear (5, 6). Current guidelines for the management of severe FMR recommend consideration of surgical intervention (repair or replacement) in symptomatic patients only following optimization of medical treatment ± cardiac resynchronization therapy (7, 8). Although annuloplasty is associated with a lower operative mortality compared to replacement, the recurrence rate of mitral regurgitation is high in patients with FMR and neither surgical repair or replacement have been shown to reduce hospitalization or death in FMR (5, 6, 9). Furthermore, nearly half of patients are deemed too high risk for surgery and therefore most patients are managed conservatively and there remains an unmet clinical need (10).Transcatheter mitral valve interventions are an emerging alternative for those at high surgical risk. These treatments are rapidly evolving with a number of novel transcatheter mitral techniques now available, many of which mimic surgical repair. Due to the complexity of the mitral valve apparatus, various techniques have been designed to target certain aspects of failure of the mitral apparatus. As FMR is predominantly a disease of the LV with failure of leaflet coaptation, the aim is to reduce the septal-lateral distance of the mitral annular plane and/or increase coaptation of the leaflets. Transcatheter annuloplasty techniques serve to reduce annular dimensions and differ from surgical techniques in that they provide the option of both direct and indirect approaches (11–15), each of which have their own potential advantages. Direct annuloplasty enables closer approximation to the mitral valve, whereas indirect annuloplasty is potentially a much simpler procedure. This mini review focuses on indirect mitral annuloplasty: anatomical considerations, patient selection, current devices, implantation techniques, and the associated clinical outcome data.

## Anatomical Considerations for Indirect Annuloplasty

**Figure 1:**
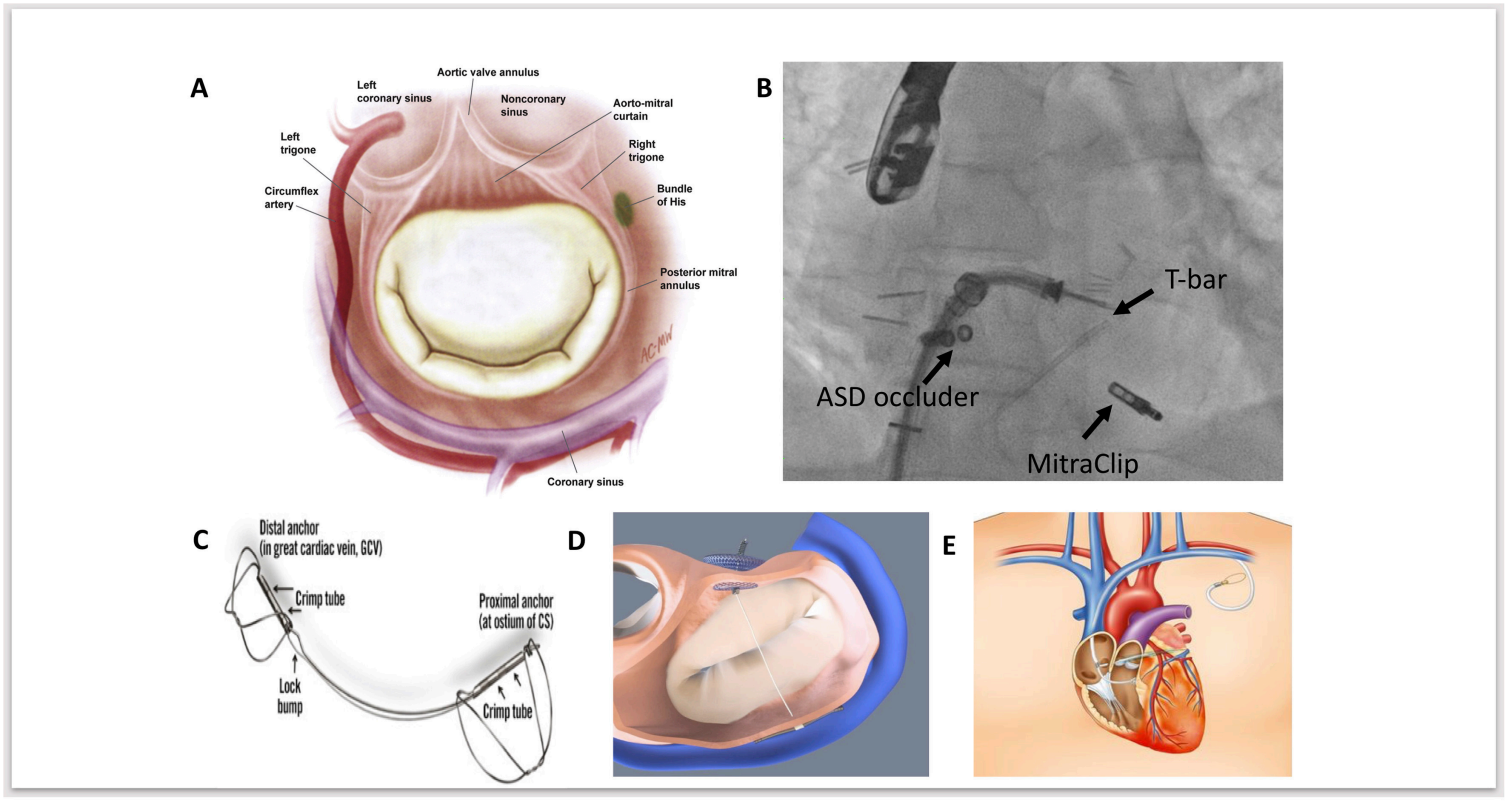
**(A)** Anatomical relationships of the mitral valve, demonstrating the close proximity of the mitral annulus, coronary sinus, circumflex artery and conduction system. Adapted and reprinted from Carpentier's Reconstructive Valve Surgery *with permission from Elsevier*. **(B)** Fluoroscopic image of MitraClip implantation as a second procedure, following a previous ARTO device; fluoroscopic landmarks for this are the Atrial Septal Defect (ASD) occluder device and the T-Bar. **(C)** The Carillon coronary sinus implant (Cardiac Dimensions) device. *Adapted and reprinted from Eurointervention, Natarajan et al, The big parade: emerging percutaneous mitral and tricuspid valve devices, 2017, with permission from Europa Digital & Publishing*. **(D)** Graphical image of the ARTO (MVRx Inc) device following deployment, with two anchors either side of the tether. In this image projection, the T-bar anchor sits inferiorly and the atrial septal anchor (occluder device) sits superiorly. *Adapted and reprinted from Eurointervention, Natarajan et al, The big parade: emerging percutaneous mitral and tricuspid valve devices, 2017, with permission from Europa Digital & Publishing*. **(E)** Graphical image demonstrating the anatomical course of cerclage annuloplasty to reduce mitral annular dimensions. Adapted and reprinted from Mitral Loop Cerclage Annuloplasty for Secondary Mitral Regurgitation, Park et al *with permission from Elsevier*.

The coronarysinus (CS) drains the majority of blood from the heart. It arises from the termination of the great cardiac vein, running through the left atrioventricular groove, emptying into the right atrium. The CS lies in close anatomical proximity to the mitral annulus (Figure 1A) (16). Indirect annuloplasty therefore utilizes the CS to exert a constraining force on the mitral annulus, thereby decreasing its septal-lateral diameter, improving leaflet coaptation and reducing the degree of mitral regurgitation. However, anatomical variation between individuals may limit the clinical efficacy of this approach. Indirect annuloplasty relies on the proximity of the CS to the mitral annulus—however, the CS is located superior to the mitral annulus in a significant number of patients and is often higher posteriorly than anteriorly (17). Furthermore, the distance between the mitral annulus and CS tends to increase in patients with dilated ventricles and severe MR (18). This could therefore explain the variation in clinical efficacy amongst different indirect annuloplasty devices.

Importantly, the circumflex coronary artery lies within close proximity of both the CS and mitral annulus. Studies have demonstrated that the vessel exhibits a deep course between the CS and mitral annulus in up to two thirds of patients (19, 20). There is therefore a theoretical risk of compression and myocardial infarction associated with indirect annuloplasty. Accurate pre-procedural imaging assessment of the venous system, coronary sinus anatomy, and mitral annular plane is essential to determine suitability and ensure appropriate patient selection prior to device implantation.

## Patient Selection for Annuloplasty Techniques

The complexity of the mitral valve apparatus necessitates patient-specific tailoring using the appropriate reparative technique because no single transcatheter technology “fits-all.” Assessment of suitability prior to annuloplasty is crucial and decision with regard to repair technique should be based on clinical and anatomical characteristics. When selecting the appropriate transcatheter therapy, it is important to first establish the primary mechanism of MR, it's severity and the imaging criteria that will predict procedural success. Traditionally, annuloplasty, either with direct or indirect percutaneous techniques are favored where annular dilatation is the predominant pathology. Surgical features of annuloplasty failure should also be taken into consideration, these include but are not limited to, increased annular dimensions (≥3.7 cm), increased systolic tenting height, complex jet(s) of mitral regurgitation and lateral wall motion abnormalities (21, 22). Furthermore anatomical considerations including the position of the CS in relation to the mitral annulus and position of the coronary arteries must also be taken into consideration. In Table 1 we summarize the clinical and echocardiographic criteria for the currently available indirect annuloplasty techniques and the comparable reduction in MR from the respective clinical trials and compare these to direct annuloplasty and edge-to-edge repair. Edge-to-edge repair may be the preferred initial therapy in FMR if the predominant mechanism of failure of coaptation is leaflet tethering or prolapse, as can be the case with ischemic MR, where leaflet tethering and annular dilatation can coexist. Percutaneous edge-to-edge repair joins the anterior and posterior leaflets using a clip, mimicking the surgical Alfieri technique and can be used in the treatment of both degenerative and FMR (25–27). Edge-to-edge repair has been shown to improve clinical outcomes in FMR with a greater benefit shown with increasing MR severity (23). Recent randomized trial data in favor of edge-to-edge repair in FMR would suggest a greater benefit in patients with severe heart failure symptoms (NYHA III-IV), larger regurgitant volume, with smaller LV end-diastolic dimensions (23, 28). However, assessment of patient suitability for edge-to-edge repair is necessary (Table 1) and increased severity of MR may necessitate more than one clip. The surgical Alfieri technique is often performed in conjunction with annuloplasty, as such there may be scope for performing combined transcatheter mitral interventions in these patients (see Figure 1B). Although this is yet to be demonstrated on a larger scale, reports of indirect or direct annuloplasty following edge-to-edge repair demonstrate reasonable outcomes. However, there is theoretical risk of mitral valve outflow obstruction with more than one device, thus more data are required if there is to be a role for this in the future.

**Table 1:** Summary of the indirect annuloplasty devices in use for functional mitral regurgitation, criteria for implant and supporting data compared with direct annuloplasty device and MitraClip.

**Technique**	**Device**	**Indication**	**Trial & design**	**Number of patients**	**Imaging & clinical inclusion criteria**	**Mean reduction in RV (mL)**	**CE mark**
Indirect annuloplasty	Carillon	Secondary/FMR with annular dilatation	AMADEUS phase I safety trial	48 (30 received device)	Moderate or severe FMR, EF <40%, NYHA class II-IV symptoms	−8.8 (6 months), *P* < 0.001	YES
			TITAN prospective non-randomized multicentre trial	53 (36 received the device)	Moderate or severe FMR, EF <40%, NYHA class II-IV symptoms despite OHFT, 6-min walk 150–450 m	−17 ± 12 (1 year), *P* < 0.001	
			REDUCE FMR sham control trial	87 device groups vs. 33 sham procedure	Moderate or severe FMR, EF <50%, NYHA class III-IV symptoms despite OHFT, LVEDD >55 mm	−7.1 vs. + 3.3 controls (1 year), *p* = 0.03	
	ARTO	Secondary/FMR with annular dilatation	MAVERIC phase 1 safety trial	11	Moderate or severe FMR, EF <40%, LVEDD >50 mm & ≤ 75 mm	−25.9 (30-days), *p* NS	NO
	Mitral Loop Cerclage System	Secondary/FMR with annular dilatation	Phase 1 trial	5	Severe FMR (RV≥ 30 mL, RF≥ 50 mL, EROA ≥ 0.20 cm^2^), NYHA class III-IV despite OHFT	−36.3 (6 months), *p* NS	NO
Direct annuloplasty	Cardioband (Edwards Lifesciences, California, USA)	Secondary/FMR with annular dilatation	Single-arm, multicenter prospective trial	31	Severe FMR despite OHFT including CRT	−11.4 (6 months), *p* = 0.063	YES
Edge to edge repair	MitraClip (Abbott Vascular, Illinois, USA)	Primary degenerative (FDA approved) and ischemic MR (secondary) with annular dilatation	COAPT Randomized controlled open label trial	302 device vs. 312 control (OHFT)	Moderate to severe FMR despite OHFT, EF 20–50%, LVEDD ≤ 70 mm NB: more than one clip or alternative to be considered if flail width>15 mm, gap>10 mm, coaptation depth >11 mm; not suitable for rheumatic or bileaflet flail	NS, however ≤moderate MR in 94.8 vs. 46.9% controls, *P* < 0.001	YES

*Nickenig et al.(11), Stone et al.(23), Goldberg et al. (24) CRT, cardiac resynchronization therapy; EF, ejection fraction; EROA, effective regurgitant orifice area; FMR, functional mitral regurgitation; LVEDD, left ventricular end diastolic diameter; NS, not specified; NYHA, New York heart association; OHFT, optimal heart failure therapy; RF, regurgitant fraction; RV, regurgitant volume*.

## Carillon Device

The Carillon coronary sinus implant (Cardiac Dimensions) is currently the only CE approved indirect annuloplasty device undergoing clinical use. The main advantage is its simplicity and safety profile, and more than 700 procedures have been performed worldwide to date (29). The Carillon device is a fixed length nitinol system that is delivered to the CS via a 9 French delivery system through the right external jugular vein (Figure 1C) (30). The device is comprised of a distal and proximal anchor. The distal anchor is deployed deep in the CS encircling the mitral annulus and traction is applied thereafter through foreshortening of the central nitinol element, thus constricting the coronary sinus by cinching the posterior peri-annular tissue and reducing mitral annular dimensions. Following confirmation of reduced mitral annular dimensions, a check angiogram is performed to ensure circumflex patency prior to final device release.

Two clinical trials of safety and feasibility have been conducted to date. The Carillon Mitral Annuloplasty Device European Union Study (AMADEUS) study successfully implanted devices in two thirds of patients selected to undergo the procedure. Patients in the AMADEUS study had only modest reductions in MR at 6-month follow up (14). The Transcatheter Implantation of Carillon Mitral Annuloplasty Device (TITAN) trial, 36 patients underwent device implantation and 17 had the device recaptured, the latter were used as a comparator group. There was no difference in the composite safety endpoint and the reduction in MR was more significant in the cohort that received the device, with an average decrease in regurgitant volume of 17 ml. This was accompanied by a significant reduction in LV systolic and diastolic dimensions at 12 and 24 months following successful implantation (31).

More recently, the outcomes of the REDUCE-FMR trial of efficacy and safety of Carillon implantation vs. sham control in patients with functional MR secondary to dilated ischemic or non-ischemic cardiomyopathy have been presented (24). The primary efficacy outcome of reduction in mitral regurgitant volume at 1 year was met (−7.1 vs. 3.3 ml; *P* = 0.03), the numerical reduction of MR was even more notable in the per protocol analysis (−12.5 vs. 1.3 ml; *p* = 0.06). Furthermore, no significant difference in major adverse cardiovascular and cerebrovascular events was demonstrated between the Carillon and sham control cohort. The CARILLON FDA trial (NCT03142152) of 450 patients randomized to the CARILLON device with optimal heart failure therapy vs. optimal heart failure therapy alone is currently open to recruitment in the United States.

## ARTO Device

The ARTO system (MVRx Inc., Belmont, CA, USA) is comprised of two anchors deployed over the lateral wall of the left atrium via the CS and in the atrial septum, connected by a tether that traverses the left atrial chamber (Figure 1D). Erglis et al. (15) Implantation is performed using transesophageal echocardiographic guidance with the patient under general anesthetic. Two venous access sites are required to deliver the device. One of two magnetic catheters is positioned in the coronary sinus over the lateral wall of the left atrium through right jugular venous access. The second magnetic catheter is positioned across the atrial septum via femoral venous access and trans-septal puncture. These two catheters are then manipulated and linked magnetically in the posterior left atrium adjacent to the posterior mitral annulus. A small puncturing wire is then used to create a connection between the two magnetic catheters. Routine catheter exchanges are performed to deliver a coronary sinus anchor (T-bar) and atrial septal anchor, connected by a suture whose length can be adjusted to reduce the anteroposterior (AP) diameter of the mitral annulus until an acceptable reduction in MR is achieved. This suture is then locked and cut.

In the first phase of the MitrAl ValvE RepaIr Clinical (MAVERIC) trial, 11 patients underwent successful device implantation with one device displacement and one pericardial effusion requiring surgical intervention. At 30-day follow up, a decrease in regurgitant volumes from 45.4 ± 15.0 ml to 19.5±10.2 ml was demonstrated with a beneficial effect on LV volumes. LV end-systolic volume index improved from 77.5 ± 24.3 ml/m^2^ to 68.5 ± 21.4 ml/m^2^, and LV end-diastolic volume index from 118.7 ± 28.6 ml/m^2^ to 103.9 ± 21.2 ml/m^2^. Mitral annular AP diameter decreased from 45.0 ± 3.3 mm to 38.7 ± 3.0 mm with an associated improvement in New York Heart Association (NYHA) functional class (32). Data at 2-year follow up demonstrated a consistent significant improvement in functional MR grade, regurgitant volumes (39.1 ± 11.6 ml vs. 14.0 ± 10.3 ml; *p* < 0.001) and reduction in mitral annular AP diameter (45.9 ± 3.1 mm vs. 39.8 ± 3.3 mm; *p* < 0.001). These changes were associated with an improvement in symptomatic status from 81.8% NHYA functional class III/IV at baseline to 60.0% NYHA functional class I/II at 2 years (33). Phase II of the MAVERIC trial is ongoing with 34 patients enrolled at 8 sites.

## Mitra Loop Cerclage System

The Mitral Loop Cerclage annuloplasty system (Tau-PNU Medical Co, Ltd.) consists of a stainless-steel tension element delivered using a multistep procedure to form a continuous loop from the coronary sinus to a basal septal perforator coronary vein and right ventricular outflow tract (Figure 1E) (13). It has a coronary sinus tricuspid bridge device (that straddles and protects the septal tricuspid leaflet and coronary conduction system) completing the loop. There is an arch-like coronary artery protection element to prevent compression of the circumflex and the device can be tensioned in real-time under echocardiographic guidance to titrate the indirect annuloplasty.

Implantation is performed under moderate sedation (transthoracic echocardiogram) or general anesthesia (transesophageal echocardiogram). Access is via 19 Fr sheaths in the left subclavian and right femoral vein. A dual lumen coronary sinus guiding catheter is introduced into the coronary sinus via the left subclavian and contrast injection used to identify a basal septal perforator vein through which a stiff tipped peripheral guidewire is introduced and used to traverse the septum into the right ventricular outflow tract before snaring into the femoral vein. The tension element is then connected to the guidewire using heat-shrink tubing and pulled into position through the CS into the interventricular septum before loop snaring of the distal guide wire tip from the femoral vein into the subclavian. Next, the bifid coronary sinus tricuspid valve bridge is advanced over the two free ends of the tension element and the coronary artery protection element positioned with diagnostic angiography. Tension is then applied to reduce the septal lateral distance and the tension device is locked and embedded in the subclavian pocket.

First in human results demonstrate successful implantation in 4 out of 5 patients. Failure to implant in one was due to unsuitable anatomy. Of those who underwent successful implantation, one patient suffered myocardial infarction and one patient died of refractory heart failure at 6 weeks. All patients demonstrated an immediate reduction in regurgitant volume. At 6-month follow up, regurgitant volume continued to decrease in the remaining 3 patients and was associated with a reduction in left atrial and LV systolic and diastolic size. Interestingly, two patients reverted to sinus rhythm at the end of the procedure (34). This was speculated to be secondary to electrical remodeling induced by the cerclage device, but may also be as a result of reduced cardiac dimensions.

## Failed Indirect Annuloplasty Devices

Coronary sinus constriction can lead to complex torsional deformation due to the complexity of the mitral annular plane and position of the CS in relation to the mitral annulus. This has unfortunately led to the failure of two previously developed devices despite encouraging early clinical safety and feasibility data (35, 36). The Viacor Percutaneous Transvenous Mitral Annuloplasty (PTMA) device comprised nitinol rods positioned in the CS to compress the posterior mitral annulus—however, device fracture in one patient led to a late, fatal coronary sinus laceration and removal from clinical use. The MONARC device (Edwards LifeSciences) was a spring-like band deployed in the coronary sinus with two self-expanding stents at either end (36). However, this device is also no longer in use due to a number of reported fractures between the band and the stents.

## Future Directions and Limitations

Functional MR is an unmet clinical need in those on maximal medical therapy but considered too high risk for conventional surgery. Annuloplasty techniques and the associated data are promising. However, annuloplasty techniques may not be suitable for all patients. Anatomical variation between individuals may limit the clinical efficacy of this approach as indirect annuloplasty relies on the proximity of the CS to the mitral annulus. Increasing LV dilatation further increases the distance between the mitral annulus and CS potentially rendering this approach ineffective. The reduction in annular dimensions from percutaneous interventions have not been as large as suggested in the surgical literature and longer-term clinical data are required to ensure safety and efficacy of these devices due to the risk of device erosion and coronary occlusion and also to assess for recurrence of MR.

**Figure 2:**
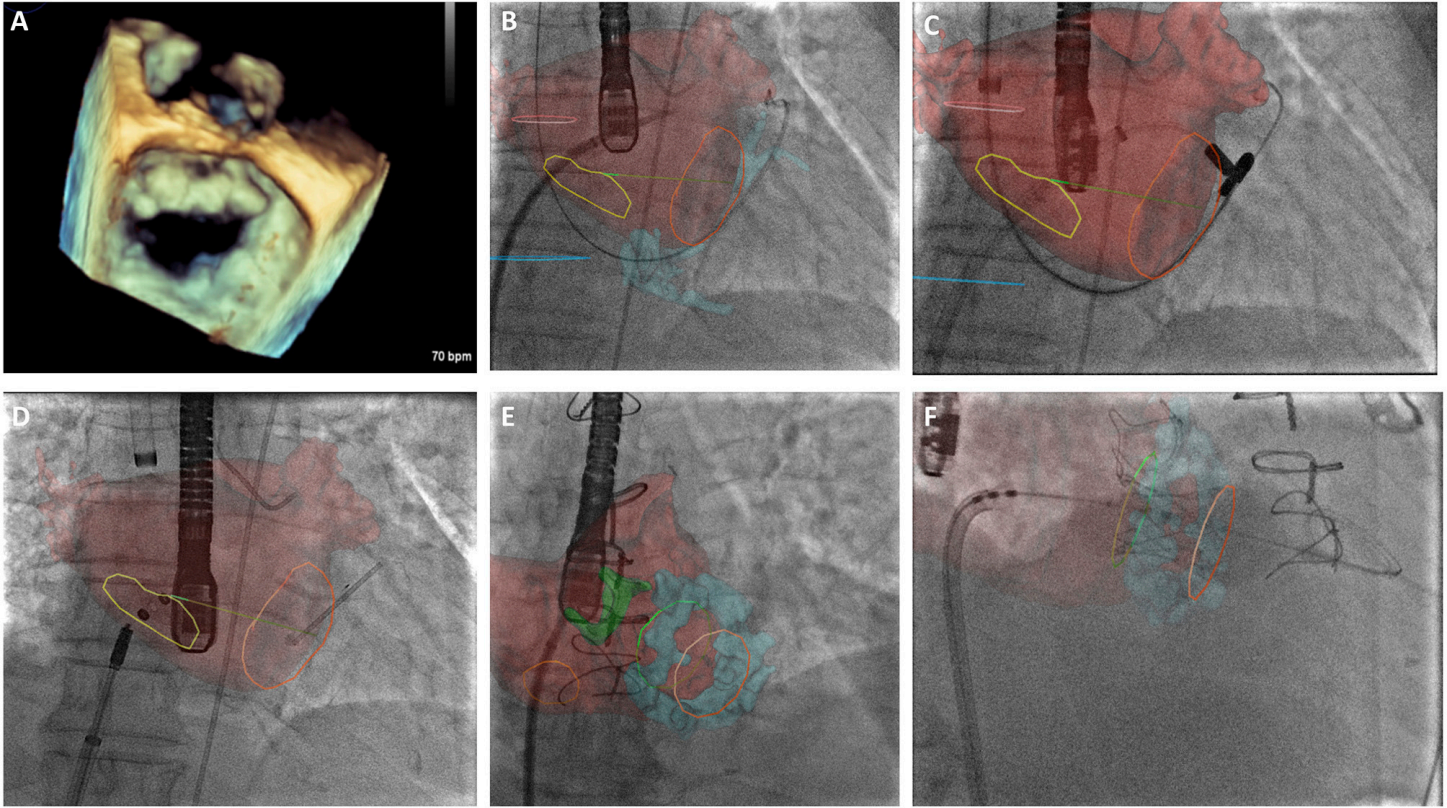
**(A)** 3-Dimensional transesophageal echocardiographic (TEE) real-time reconstruction of the mitral valve annulus and leaflets as a preliminary investigation to determine anatomical suitability for transcatheter mitral intervention. **(B)** CT overlay with real-time image fusion to demonstrate the optimal site for trans-septal puncture for ARTO case. Yellow line delineates inter-atrial septum, left atrium is superimposed in red. **(C)** CT overlay with real-time image fusion during magnet positioning during ARTO case **(D)** real-time image fusion demonstrating T-bar and atrial septal defect (ASD) occluder device device positioning relative to mitral annulus (orange circle) and inter-atrial septal markers (yellow circle), respectively. **(E)** CT overlay with real-time image fusion during transeptal puncture for transcatheter mitral valve implantation in mitral annular calcification identifying interatrial septum (orange circle), atrial anatomy (red) and mitral annular calcification (blue), aortic bioprothesis is also delineated (green). **(F)** CT overlay with real-time image fusion to facilitate transcatheter mitral valve in MAC positioning, atrial anatomy (red) and mitral annular calcification (blue) are visualized in addition to the superior (green) and inferior markers (orange).

The recent results of the MITRA-FR and COAPT trial have helped define a patient population in whom there is potential benefit from MitraClip implantation (Table 1). The surgical Alfieri technique is frequently performed in conjunction with annuloplasty, and there may be scope for performing combined transcatheter mitral repair in these patients, however this is yet to be demonstrated on a larger scale. It would be advisable for centers providing transcatheter mitral interventions to be trained in a number of techniques so as to appropriately select the patient cohort that would benefit from a specific technique. An increased appreciation of the mitral valve apparatus will no doubt aid development of further novel mitral technologies and second and third generation devices are anticipated to improve procedural safety and success rates. Such devices will require large scale clinical validation and Heart Team involvement will be essential to determine patient suitability. Due to the complexity of the mitral valve apparatus, Heart Team decision making will require evaluation of patient-specific anatomical characteristics using novel imaging techniques, including 3D TEE and CT image fusion (Figures 2A–F). This will aid decision-making and guide periprocedural planning and implantation to ensure successful procedures with minimal complications.

## Conclusions

An increase in anteroposterior (AP) mitral annular diameter is the common final pathway in the development of functional MR and associated with worsening clinical outcomes in heart failure and post myocardial infarction. Shortening of the AP dimension is therefore critical to alleviating MR. The aim of transcatheter mitral repair is to balance the increase in peri-procedural safety (reduced risk) with a sufficient reduction in MR for it to be effective. Annuloplasty, both direct and indirect, leaflet repair and chordal repair are all viable options based upon well-established surgical techniques and a combination of these approaches may provide the most effective resolution of MR. Current predictors of MR recurrence following surgical repair include baseline LV end-diastolic diameter >65 mm, posterior mitral leaflet angle >45 degrees and mitral coaptation depth >10 mm (37). However, the relevance of these for the success of percutaneous interventions remains unknown. Furthermore, there are numerous challenges to effective treatment of MR, including anatomical variation and the complexity of the mitral valve apparatus, imaging constraints and currently available technologies. There remain important considerations when determining suitability for percutaneous mitral valve interventions, including appropriate patient selection (moderate vs. severe MR, normal vs. impaired LV function) and choice of device based on anatomical characteristics. Although further work is required to ensure safety and durability of these devices, increased understanding of the true incidence, natural history and pathophysiology of MR, will enable better targeted device therapy in this cohort.

## Author Contributions

SR manuscript conception, design, and critical revision. BP manuscript critical revision. RR manuscript critical review and revision. TP manuscript conception, design and critical revision. HA critical revision of the manuscript. CA critical revision of the manuscript.

### Conflict of Interest Statement

The authors declare that the research was conducted in the absence of any commercial or financial relationships that could be construed as a potential conflict of interest.
